# Exploring the diversity of uncommon oral yeast species and associated risk factors among substance abusers in southwestern Iran

**DOI:** 10.1038/s41598-024-52105-4

**Published:** 2024-01-22

**Authors:** Aynaz Ghojoghi, Sadegh Khodavaisy, Ali Zarei Mahmoudabadi, Eisa Nazar, Mahnaz Fatahinia

**Affiliations:** 1 Department of Medical Mycology, School of Medicine, Ahvaz Jundishapur University of Medical Sciences, Ahvaz, Iran; 2 Cellular and Molecular Research Center, Medical Basic Sciences Research Institute, Ahvaz Jundishapur University of Medical Sciences, Ahvaz, Iran; 3 Department of Medical Parasitology and Mycology, School of Public Health, Tehran University of Medical Sciences, Tehran, Iran; 4 Research Center for Antibiotic Stewardship and Antimicrobial Resistance, Tehran University of Medical Sciences, Tehran, Iran; 5 Infectious and Tropical Diseases Research Center, Health Research Institute, Ahvaz Jundishapur University of Medical Sciences, Ahvaz, Iran; 6 Psychiatry and Behavioral Sciences Research Center, Addiction Institute, Mazandaran University of Medical Sciences, Sari, Iran

**Keywords:** Microbiology, Fungi

## Abstract

Yeast species are a group of coexistent microorganisms in the oral cavity that can cause opportunistic infections in vulnerable individuals, including addicts. This study aimed to identify the yeast species profile responsible for oral yeast colonization (OYC) and the associated risk factors in patients with substance use disorder (SUD) in Ahvaz, Iran. Oral samples were collected from drug users hospitalized in 12 addiction treatment centers, and the related clinical information was mined. Oral yeast species were identified using 21-plex PCR and sequencing of the internal transcribed spacer region (ITS1-5.8S-ITS2). A total of 244 yeast strains were identified from 245 individuals with substance abuse. *Candida albicans* was the most common species (37.7%) and non-*albicans Candida* was responsible for 57.7% of OYC, primarily *C. dubliniensis* (33.2%) and *C. glabrata* (11.9%). Moreover, uncommon oral yeasts constituted 5.3% of species, including *Saccharomyces cerevisiae*, *Clavispora lusitaniae*, *Pichia kluyveri*, *Geotrichum candidum*, *Magnusiomyces capitatus*, *Hanseniospora opuntiae*, *Wickerhamomyces subpelliculosus*, *Trichosporon asahii,* and *Aureobasidium pullulans*. Importantly, OYC exhibited associations with such factors as duration of drug use, daily drug consumption rate, opioid utilization, oral drug administration, and the Diagnostic and Statistical Manual of Mental Disorders, Fifth Edition (DSM-5) score. The present study is the pioneering investigation revealing the prevalence and diversity of oral yeast species, along with associated risk factors, in individuals with SUD in southwestern Iran. Furthermore, it underscores the importance of developing efficient and cost-effective diagnostic methods tailored for resource-constrained settings.

## Introduction

The incidence of oral fungal infections has increased over the past few decades, which can cause symptoms ranging from mild oral disease to severe systemic infections, particularly in immunocompromised individuals^1^. Although the majority of yeast species isolated from the oral cavity are due to *Candida albicans*, infections due to non-*albicans Candida* (NAC) species, including those with resistance to azole antifungals, are progressively on the rise^2,3^. This shift in the prevalence of *C. albicans* to NAC species is worrisome. In addition, there has been a recent surge in reported cases of infections caused by uncommon yeast species such as *Trichosporon*, *Rhodotorula*, *Geotrichum*, *Pichia*, and *Saccharomyces* spp.^4,5^, as well as other rarely encountered species in the human oral cavity^6^. Several underlying factors contribute to the development of oral yeast infections, including diabetes, immunodeficiency syndrome, soft tissue injury, loss of natural defensive barriers, use of artificial teeth, and addiction^7^.

Addiction is a persistent condition characterized by the compulsive use of drugs despite negative consequences, which presents a major threat to both individuals and societies. Today, addiction is considered as a major public health concern due to its association with a range of adverse health outcomes and infections^8^. People with substance use disorders (SUD) are more vulnerable to oral yeast infections due to factors such as compromised oral hygiene, immunosuppression, malnutrition, and interactions between substances and oral microorganisms^9,10^. In addicted individuals, particularly those with compromised immune systems, these factors can not only harm mucosal immune defenses but also disrupt the physiological and microbial ecology of the oral environment. This can lead to chronic dry mouth and reduced salivation, which promote the colonization of yeast species in the mouth. Consequently, systemic infections associated with significant morbidity and mortality rates may occur^11^.

Traditionally, the widely available methods for identifying yeast species in clinical laboratories are phenotypic and biochemical assays, but these methods can be labor-intensive, time-consuming, and prone to errors, particularly for unusual yeast species^12^. Although ribosomal DNA sequencing is considered to be the gold standard technique for the identification of yeast species, this technique is expensive and thus not widely utilized in routine laboratories^13^. On the other hand, there are only a few polymerase chain reaction (PCR) based techniques available that can accurately target a comprehensive list of opportunistic yeast species^14^. Recently, the 21-multiplex PCR assay has proven to be a successful rapid technique for identifying opportunistic yeast species. An elegant study showed that 21-plex PCR assay was capable of identifying the causative agents of 95% of yeast-associated infections in a stepwise manner, using PCR product size^12^. Since utilizing the 21-plex PCR test to identify common and unusual yeasts can decrease processing times and expenses, this approach could be helpful for use in developing countries.

Accordingly, this study was conducted to explore the prevalence and diversity of oral yeast species among individuals with SUD in Ahvaz, southwestern Iran. The innovative use of the 21-plex PCR technique expedites yeast species identification and provides a cost-effective alternative, especially in resource-constrained settings. The research can significantly enhance our understanding of oral *Candida* spp. and uncommon oral yeasts in individuals with SUD by introducing some novel data. Moreover, the findings can help identify key risk factors, including demographic characteristics and drug-related factors, contributing to oral yeast colonization (OYC). The outcomes might offer valuable insights for effective management and appropriate intervention strategies in this vulnerable population.

## Materials and methods

### Study design and patient characteristics

This study included a total of 245 participants (183 males and 62 females) hospitalized in 12 addiction treatment centers in Ahvaz, southwestern Iran. The study protocol was approved by the Research Ethics Committee of Ahvaz Jundishapur University of Medical Sciences, Iran (code: IR. AJUMS.MEDICINE. REC.1400.047). All participants were included in the study after signing an informed consent form prior to inclusion in the study. Oral samples were collected between 27 December, 2021 and 19 July, 2022. The patients’ demographic details along with the drug use data [type of drug, method of use, duration of use, the Diagnostic and Statistical Manual of Mental Disorders (DSM-5) criteria, etc.] were collected at the time of enrollment. The inclusion criteria were as follows: being drug addicts receiving inpatient care at addiction treatment centers, providing an informed consent to participate in the study, and having medical records available at the centers.

### Initial identification of yeasts

Sampling of the internal surfaces of the entire oral cavity was performed using a sterile cotton swab. The obtained oral samples were promptly subjected to cultivation on CHROMagarTM *Candida* medium (CHROMagarTM, Pioneer, Paris, France) plates and then incubated at 35 °C for 48 to 72 h. Afterwards, the yeasts were differentiated according to the morphology and color of their colonies, and the number of colonies was counted based on their colony forming units (CFUs) per swab. Subjects who had a CFU swab count of > 10 were classified into the OYC^15,16^. The yeast isolates obtained from the chromogenic medium were subsequently transferred into two sets of microtubes each filled with sterile distilled water. The microtubes served as long-term sources for the isolates and were kept under both room temperature and refrigerated condition.

### DNA extraction

The yeast isolates were subcultured on Sabouraud dextrose agar (Lifoilchem, Roseto degli Abruzzi, Italy) containing chloramphenicol for 48 h. To initiate DNA extraction, one full loop of the freshly grown yeast colonies was transferred into 2 ml screw cap tubes. The tubes were then supplemented with 100 μl glass beads, 300 μl of lysis buffer (composed of 200 mM Tris–HCl pH 8, 25 mM EDTA, 250 mM NaCl, and 0.5% sodium dodecyl sulfate), and 300 μl of phenol chloroform/isoamyl alcohol. After vortexing the suspensions for 2 min, they were centrifuged for 5 min at 10,000 rpm. The resulting supernatant was then carefully transferred to a new 1.5 ml tube, followed by the addition of an equal volume of isopropanol, 300 µl of chloroform, and 0.1 volume of 3 M sodium acetate (pH 5.2). Next, the solutions were briefly vortexed and incubated at − 20 °C for 10 min. This was followed by centrifugation at 12,000 rpm for 15 min. The resulting precipitate was washed with ice-cold 70% ethanol and centrifuged at 12,000 rpm for 15 min. The supernatant was then removed, and the pellet was air-dried before being dissolved in 50 µl of distilled water. The yield and purity of the extracted DNA were assessed using a nanophotometer by measuring the absorbance at 260 nm and 280 nm. The DNA samples were stored at − 20 °C for subsequent molecular identification procedures.

### Molecular assays

The identification of yeasts was carried out using the 21-plex PCR method, following a protocol outlined by Arastehfar et al.^12^. This technique includes three multiplex PCR reactions, with the first identifying the most common *Candida* species, the second targeting uncommon *Candida* species, and the third multiplex test identifying the most clinically significant basidiomycete yeasts like *Geotrichum*, *Trichosporon*, *Cryptococcus*, and *Rhodotorula*. After amplification, the PCR products and a 100 bp ladder were separated on a 2% agarose gel for 60 min at 100 V. The gel was stained with SYBR green (Parstous, Iran) and examined under UV light to identify the yeast species by discerning differences in the fragment size of the PCR products^12^. The rDNA sequencing was used to identify yeast species that were not identified by the 21-plex PCR technique. These regions of the yeasts were amplified using the ITS1 and ITS4 primers, with the following sequences: ITS1 5′ TCCGTAGGTGAACCTGCGG 3′ and ITS4 3′TCCTCCG CTTATTGATATGC^17^. Bidirectional chain Terminated Sanger sequencing using referenced primers were performed. The resulting nucleotide sequences were compared to known sequences in the NCBI GenBank database using the BLAST online tool. The species of each isolate was assigned based on the sequence with the highest similarity, and then the results were deposited to the GenBank.

### Statistical analysis

The Chi-square and Fisher’s exact tests were employed to compare the frequency distribution of qualitative variables among patients with and without OYC. Moreover, the independent t-test was employed to compare the means of quantitative variables between the two groups of patients (with OYC versus without OYC). Further analysis was performed using multiple logistic regression (MLR) model to investigate the factors associated with having OYC among the subjects. The variables with a P-value < 0.25 in the univariate LR model were entered into the MLR model. In addition, we compared the overall discriminative capacity of the variables regarding OYC by using the area under the receiver operating characteristic curve (AUC). The receiver operating characteristic (ROC) curves were used to assess the ability of the MLR model to predict OYC among the subjects. All analysis was performed in the SPSS v.22 (SPSS Inc., Chicago, IL, United States) at the significant level of 0.05.

## Results

### Distribution of yeast species causing OYC

In total, we diagnosed 244 strains of yeasts and yeast-like fungi from 245 substance abusers (Table 1). *C. albicans* was the most prevalent yeast species (37.7%), and NAC species caused 57.7% of OYC. The most common species among the NAC isolates were *C. dubliniensis* and *C. glabrata,* with prevalence rates of 33.2% (n = 81) and 11.9% (n = 29), respectively.

**Table 1:** Frequency of yeast species isolated from the oral cavity of addicted patients (n = 245).

Species	Number of isolates	Isolation rate by species, %	Isolation rate by patients, %	Colony color on CHROMagar
*Candida albicans*	92	37.70	37.55	Green
*Candida dubliniensis*	81	33.20	33.06	Green
*Candida glabrata*	29	11.89	11.84	Mauve
*Pichia kudriavzevii (Candida krusei)*	10	4.10	4.08	Pink/fuzzy
*Candida parapsilosis*	10	4.10	4.08	Pink
*Candida tropicalis*	5	2.05	2.04	Metallic blue
*Kluyveromyces Marxianus (Candida kefyr)*	4	1.64	1.63	Pink
*Saccharomyces cerevisiae*	2	0.82	0.82	Purple
*Clavispora lusitaniae (Candida lusitaniae)*	2	0.82	0.82	Purple and white
*Pichia kluyveri*	2	0.82	0.82	Purple/fuzzy
*Geotrichum candidum*	2	0.82	0.82	White
*Magnusiomyces capitatus*	1	0.41	0.41	White and pink
*Hanseniospora opuntiae*	1	0.41	0.41	Purple
*Wickerhamomyces subpelliculosus*	1	0.41	0.41	Pink
*Trichosporon asahii*	1	0.41	0.41	White to lavender
*Aureobasidium pullulans*	1	0.41	0.41	Cream

In this study, 5.3% of the identified species (13/244) were rare oral yeasts, including *Saccharomyces cerevisiae* (2/244; 0.82%), *Clavispora lusitaniae* (2/244; 0.82%), *Pichia kluyveri* (2/244; 0.82%), *Geotrichum candidum* (2/244; 0.82%), *Magnusiomyces capitatus* (1/244; 0.41%), *Hanseniospora opuntiae* (1/244; 0.41%), *Wickerhamomyces subpelliculosus* (1/244; 0.41%), *Trichosporon asahii* (1/244;0.41%), and *Aureobasidium pullulans* (1/244; 0.41%). The data also demonstrated that more than one *Candida* spp. was isolated in 64 patients (38.8%) (Table 2). The most common co-colonization was a combination of *C. albicans* and/or *C. dubliniensis* with NAC species.

**Table 2:** Distribution of co-colonization with different yeast species in 165 patients with OYC.

Co-colonization of species	Patients, *n* (%)
*C. albicans* and* C. dubliniensis*	13 (7.9)
*C. albicans* and *C. glabrata*	11 (6.7)
*C. dubliniensis* and *C. glabrata*	6 (3.7)
*C. albicans* and* C. dubliniensis & C. glabrata*	4 (2.5)
*C. dubliniensis* and* C. parapsilosis*	3 (1.8)
*C. dubliniensis* and* C. krusei*	3 (1.8)
*C. albicans* and* C. dubliniensis & C. tropicalis*	2 (1.2)
*C. albicans* and* C. krusei*	2 (1.2)
*C. dubliniensis* and* C. kefyr*	2 (1.2)
*C. albicans* and* C. dubliniensis* and* S. cerevisiae* and* C. glabrata*	1 (0.6)
*C. dubliniensis* and* C. parapsilosis* and* C. glabrata* and* C. tropicalis*	1 (0.6)
*C. albicans* and* C. dubliniensis* and* C. krusei*	1 (0.6)
*C. albicans* and* C. dubliniensis* and* W. subpelliculosus*	1 (0.6)
*C. dubliniensis* and* C. krusei* and* C. lusitaniae*	1 (0.6)
*C. dubliniensis* and* C. krusei* and* C. parapsilosis*	1 (0.6)
*C. albicans* and* C. kefyr* and* C. parapsilosis*	1 (0.6)
*C. albicans* and* S. cerevisiae*	1 (0.6)
*C. albicans* and* T. asahi*	1 (0.6)
*C. albicans* and* C. kefyr*	1 (0.6)
*C. albicans* and* G. candidum*	1 (0.6)
*C. albicans* and* C. tropicalis*	1 (0.6)
*C. albicans* and* C. parapsilosis*	1 (0.6)
*C. dubliniensis* and* G. candidum*	1 (0.6)
*C. dubliniensis* and* P. kluyveri*	1 (0.6)
*C. dubliniensis* and* H. opuntiae*	1 (0.6)
*C. dubliniensis* and* C. tropicalis*	1 (0.6)
*C. krusei* and* P. kluyveri*	1 (0.6)
Total	64 (38.8)

**Figure 1:**
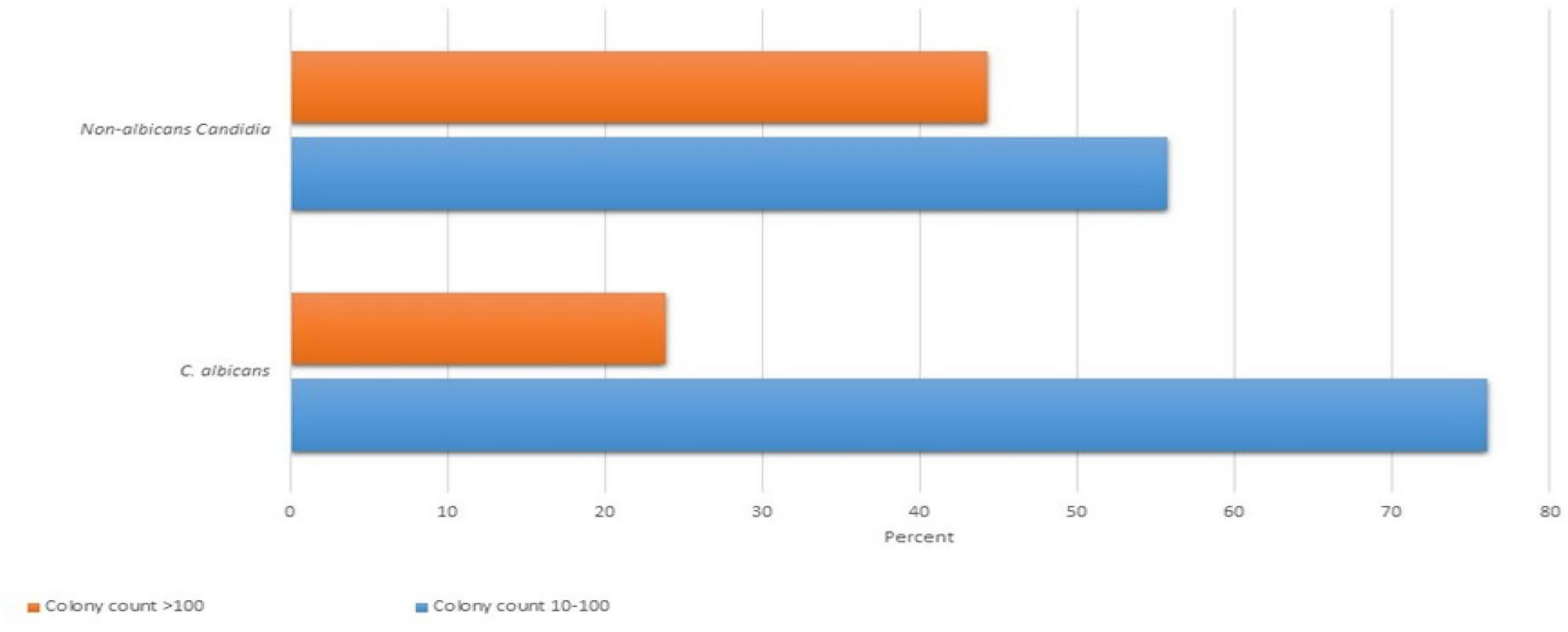
Comparing the frequency distribution of colony counts between *Candida albicans* and NAC species among individuals with OYC (P = 0.002).

Figure 1 compares the number of colonies between *C. albicans* and NAC species in individuals with OYC. The results of the chi-square test indicated a statistically significant difference in the frequency distribution of colony counts between *C. albicans* and NAC species in individuals with OYC (P < 0.05).

**Figure 2:**
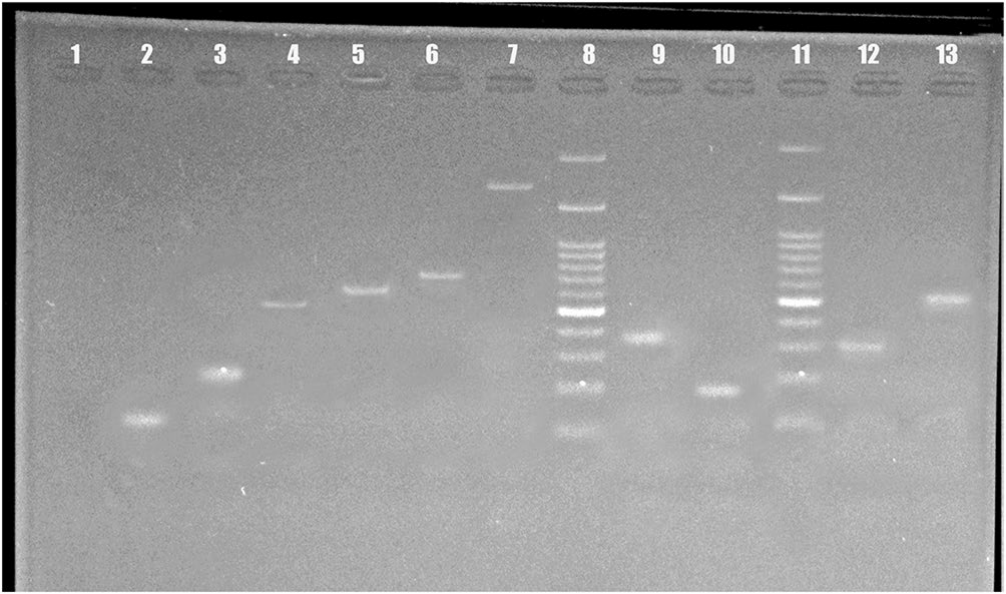
Electrophoretic pattern of 21-plex PCR products. Numbers 1 to 13 encompass the following: negative control, *Candida tropicalis, Candida glabrata, Candida parapsilosis, Candida albicans, Candida dubliniensis, pichia kudriavzevii (Candida krusei),* Marker*, Clavispora lusitaniae (Candida lusitaniae), Kluyveromyces marxianus (Candida kefyr),* Marker*, Geotrichum candidum,* and *Trichosporon asahii.*

Our data also showed that 236 yeast isolates (96.7%) were correctly identified by 21-plex PCR. This technique successfully identified all *Candida* species, as well as certain rare yeast species such as *C. lusitaniae*, *G. candidum*, and *T*. *asahii* (Fig. 2). The remaining eight unknown isolates were subsequently identified and confirmed at the species level using rDNA sequencing, revealing the presence of *S. cerevisiae*, *P. kluyveri*, *M. capitatus*, *H. opuntiae*, *W. subpelliculosus*, and *A. pullulans*. The sequences were deposited in the GenBank database with the accession numbers OQ184727, OR244181, and OQ770376-82.

### Demographic factors and presence of OYC

Out of the 245 drug abusers included in the study, 165 patients (67.30%) were diagnosed with OYC. The demographic characteristics of patients are summarized in Table 3. The mean age of the patients with and without OYC was 35.02 ± 10.39 and 30.39 ± 7.42 years, respectively. The independent t-test revealed a statistically significant difference between the mean age and presence of OYC (P < 0.05). Among the infected patients, 50 (80.60%) were females and 115 (62.80%) were males, and the chi-square test showed a statistically significant association between gender and OYC (P < 0.05). Out of the 13 cases of uncommon yeast species, 3 species (23%), including *C. lusitaniae*, *G. candidum*, and W*. subpelliculosus*, were identified in females. The remaining 10 uncommon yeast isolates (77%) were observed in males. The majority of OYC cases were observed in individuals with underlying diseases (81.80%), and a statistically significant association was found between this factor and the occurrence of OYC (P < 0.05). The results of the Chi-square test reveal a significant relationship between gender and underlying diseases (P < 0.05). Of the 62 women with OYC, 21 had underlying diseases, while among the 183 men with OYC, 23 had underlying diseases. Although the prevalence of OYC was greater among cigarette users (68.40%) than non-cigarette users (37.50%), there was no statistically significant difference in the prevalence of OYC between the two groups (P > 0.05). In addition, there was no significant association between marital status, location, education, occupation, and the occurrence of OYC (P > 0.05).

**Table 3:** Comparison of the frequency of socio-demographic characteristics between patients with and without OYC.

Variables		OYC^#^		P-value
		No	Yes	
Age	Mean $\pm$ SD	30.39 $\pm$ 7.42	35.02 $\pm$ 10.39	< 0.001
	$\le$ 50	80 (34.50)	152 (65.50)	0.006*
	> 50	0 (0.00)	13 (100.00)	
Gender	Female	12 (19.40)	50 (80.60)	0.01*
	Male	68 (37.20)	115 (62.80)	
Underlying disease	No	72 (35.80)	129 (64.20)	0.02*
	Yes	8 (18.20)	36 (81.80)	
Marital status	Single	42 (35.30)	77 (64.70)	0.08
	Married	33 (35.10)	61 (64.90)	
	Divorced/widowed	5 (15.60)	27 (84.40)	
Location	Urban	73 (33.20)	147 (66.80)	0.60
	Rural	7 (28.00)	18 (72.00)	
Education	Under diploma	65 (32.20)	137 (67.80)	0.72
	Diploma and higher	15 (34.90)	28 (65.10)	
Job	Housewife	5 (27.80)	13 (72.20)	0.42
	Employee/retired	3 (60.00)	2 (40.00)	
	Other	72 (32.40)	150 (67.60)	
Cigarette use	No	5 (62.50)	3 (37.50)	0.11
	Yes	75 (31.60)	162 (68.40)	

^#^Values are reported as frequency (percent) or mean ± SD.

*Significant at the level of 0.05.

### Factors related to drugs and presence of OYC

Table 4 presents the relationship between the consumption methods and the type of drugs with the occurrence of OYC. Based on the findings, the highest prevalence rates of OYC were related to injection (81.40%) and oral (81.40%) methods of drug consumption (P < 0.05). We also observed that OYC was most frequently observed in individuals who used stimulants (77.20%) and opioids (75.20%) (P < 0.05).

**Table 4:** Evaluating the association between methods of drug consumption and type of drug with occurrence of OYC.

Variables		OYC infection		P-value
		No	Yes	
Methods of drug consumption				
Oral	No	64 (40.30)	95 (59.70)	0.001*
	Yes	16 (18.60)	70 (81.40)	
Injection	No	72 (35.60)	130 (64.40)	0.03*
	Yes	8 (18.60)	35 (81.40)	
Smoking	No	12 (46.20)	(53.80)	0.12
	Yes	68 (31.10)	151 (68.90)	
Type of drugs				
Stimulant	No	49 (45.00)	60 (55.00)	< 0.001*
	Yes	31 (22.80)	105 (77.20)	
Hallucinogen	No	59 (33.70)	116 (66.30)	0.57
	Yes	21 (30.00)	49 (70.00)	
Opioid	No	43 (44.80)	53 (55.20)	0.001*
	Yes	37 (24.80)	112 (75.20)	
Other	No	74 (33.60)	146 (66.40)	0.33
	Yes	6 (24.00)	19 (76.00)	

*Significant at the level of 0.05.

**Figure 3:**
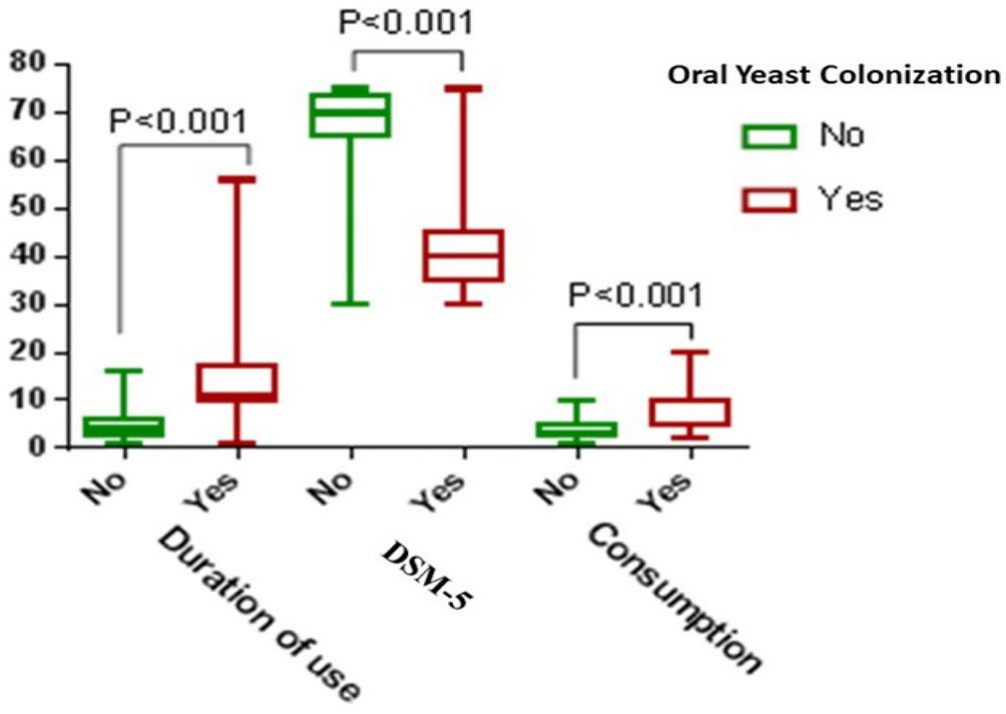
Comparing the mean of DMS-5, consumption, and duration of use between patients with and without OYC.

In addition, the analysis using independent t-tests demonstrated significant differences in the mean duration of drug use/year (4.86 ± 3.08 vs. 13.02 ± 7.68), DSM-5 score (64.25 ± 13.85 vs. 44.00 ± 13.24), and drug consumption rate/day (4.53 ± 2.10 vs. 8.10 ± 2.97) for individuals without and with OYC, respectively (Fig. 3).

### Comparing the predictability of variables with ROC curves

The ROC analyses showed that the duration of drug use (AUC = 0.8587, P < 0.001) and drug consumption rate/day (AUC = 0.8164, P < 0.001) had higher predictive power for OYC than DSM-5 score (AUC = 0.1844, P < 0.001) (Fig. S1). Furthermore, stimulant use (AUC = 0.6244, P < 0.001) had a higher predictive power for OYC than opioid use (AUC = 0.6081, P = 0.001), hallucinogen use (AUC = 0.5172, P = 0.57), and other types of drug use (AUC = 0.5201, P = 0.33) (Fig. S2). Furthermore, substance use variables associated with different consumption methods (such as oral, injection, and smoking) exhibited similar predictive power for characterizing OYC (Fig. S3).

### Factors associated with OYC using MLR analysis

The factors associated with colonization were subjected to MLR analysis. According to the results, duration of drug use (OR: 1.34; 95% CI 1.15, 1.57), drug consumption rate/day (OR: 1.47; 95% CI 1.16, 1.86), DSM-5 score (OR: 0.91; 95% CI 0.88, 0.94), opioid use (OR: 3.27; 95% CI 1.06, 10.10), and oral drug use (OR: 6.96; 95% CI 1.71, 28.26) were significantly associated with the occurrence of OYC (P < 0.05). However, other variables did not have a statistically significant association with OYC (P > 0.05) (Table 5).

**Table 5:** Determining the factors associated with OYC among addicted patients using multiple logistic regression model.

Variables (references)	OR^#^ (%95 CI)	P-value
Duration of drug use	1.34 (1.15, 1.57)	< 0.001*
Drug consumption rate per day	1.47 (1.16, 1.86)	0.001*
DMSIV score	0.91 (0.88, 0.94)	< 0.001*
Stimulant (no)	–	–
Yes	1.34 (0.43, 4.19)	0.60
Opioid (no)	–	–
Yes	3.27 (1.06, 10.10)	0.03*
Smoking (no)	–	–
Yes	3.28 (0.32, 33.44)	0.31
Oral use (no)	–	–
Yes	6.96 (1.71, 28.26)	0.007*
Injection use (no)	–	–
Yes	2.34 (0.47, 11.63)	0.29
Cigarette use (no)	–	–
Yes	2.86 (0.52, 15.68)	0.22

*OR* odds ratio.

^#^Adjusted by age, gender, marital status, and underlying disease.

*Significant at the level of 0.05.

**Figure 4:**
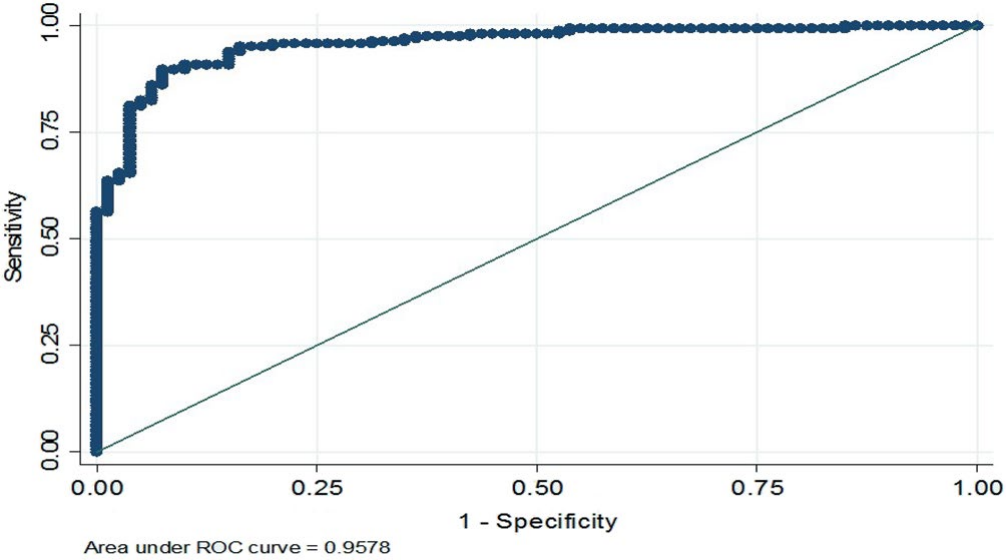
Evaluating the predictive power of the final MLR model using the ROC curve.

The area under the ROC curve of the final MLR model was 0.9578, indicating its high predictive power (Fig. 4).

## Discussion

Substance use disorders are associated with numerous medical, psychiatric, and economic problems, creating a significant burden for affected individuals and society^18^. To date, limited studies have investigated the prevalence of yeast species and the related factors in addicted individuals. To address this gap, we conducted this study in southwestern Iran and identified a noteworthy rate of OYC (67.3%) among drug abusers. Similarly, a study conducted in northeast Iran examined 83 oral samples from individuals with addiction and detected oral candidiasis in 61.8% of patients^19^. In another study conducted in central Iran, PCR results from oral samples showed a high frequency (69.8%) of *Candida* species among drug abusers^17^. In a recent study by Navabi et al., the prevalence rate of oral *candida* fcolonization was 38.7% among opium users and cigarette smokers in southeastern Iran^20^. Conversely, Khalili et al. reported a low prevalence (7.94%) of oral candidiasis in individuals using cigarettes, tobacco, alcohol, and opium in Rafsanjan, a region in southeastern Iran^21^. In another study conducted in Spain, Sheth et al. demonstrated that around 35% of volunteers (37 individuals) harbored *Candida* species in their oral cavities^22^. The microbiological analysis conducted by Hadzic et al. in Bosnia and Herzegovina confirmed a frequency of *Candida albicans* (43%) among psychoactive substance addicts and noted an elevation in NAC, irrespective of the type of addiction (34%)^23^. This variation might be due to differences in geographical locations, certain characteristics of the individuals (such as age, oral hygiene, or the presence of dentures), different sample collection methods, and diverging identification techniques.

Our data concur with our previous findings that addiction may be a contributing factor to the high occurrence of yeast species in the oral cavity. The current evidence indicates that addiction can lead to a compromised local and systemic immune system, thereby creating a conducive environment for pathogen acquisition and the development of oral yeasts^24,25^. Opiates, for instance, can directly impact the immune system by binding to opioid receptors on immune cells, resulting in reduced phagocytosis and chemotaxis^26^. On the other hand, opium addiction significantly disrupts the cytokine network, creating a conducive environment for fungal infections^9,26^. Furthermore, drug-induced vasoconstriction and reduced saliva production in individuals with addiction can elevate the increase of OYC in the oral cavity^27^. Poor oral and dental hygiene, communal living in drug addiction camps, and low economic and cultural status are important contributing factors associated with an increased risk of OYC in this population^28^. It should be noted that diagnostic techniques may also impact the prevalence of yeast species.

In this study, the combination of 21-plex PCR and rDNA sequencing proved to be a powerful technique for accurately identifying yeasts species from the oral cavity. In a previous Iranian study that evaluated candidemia in pediatric patients, 95.7% of the species identified were in the target list of the 21-plex PCR^29^. A recent study demonstrated that the 21-plex PCR assay was 100% consistent with MALDI-TOF MS^12^, while another study found that the 21-plex PCR exhibited a higher degree of accuracy for yeast species compared to the Vitek 2 system^30^.

We diagnosed 244 species of yeasts and yeast-like fungi from 245 substance abusers in this research. The most frequently isolated yeast species included *C. albicans*, which accounted for 37.7% of the isolates, followed by *C. dubliniensis* (33.2%) and *C. glabrata* (11.8%). It is worth noting that, consistent with our findings, *C. dubliniensis* was the most prevalent species isolated among NAC species in the oral specimens of individuals with SUD in different regions^23,31^. In the past, *C. dubliniensis*, due to its morphological resemblance, was frequently misidentified as *C. albicans* using traditional methods. However, accurate differentiation between the two species can now be achieved through the implementation of precise molecular methods^15^. The cause for the heightened prevalence of this species in drug-affected patients remains a matter of debate.

The present study indicated that NAC species predominantly contributed to OYC in addicted people (57.7%). Hadzic et al. showed that the abuse of psychoactive substances influences the prevalence of *C. albicans* and NAC species of oral *Candida*. According to these findings, psychoactive substances (opiates and alcohol) can lead to an increase in oral *Candida dubliniensis* regardless of the type of addiction^23^. Additionally, recent studies suggest that the rise in NAC species may be associated with factors such as immune system status, age, antifungal drug resistance, healthcare facilities, broad-spectrum antibacterial agents, and geography^3,32^. Interestingly, we found that 38.8% of patients were colonized with multiple yeast species, which contrasts with other studies involving different populations that reported lower rates of multiple colonization^33,34^. This difference in results could be attributed to the techniques employed in our study and the favorable oral cavity conditions in individuals with addiction disorders that promote yeast growth.

In our investigation, uncommon yeast species accounted for 5.3% of the total frequency. The 21-plex PCR method successfully identified various *Candida* species and rare yeasts, such as *C. lusitaniae*, *G. candidum*, and *T. asahii*. For unidentified isolates, including *S. cerevisiae*, *P. kluyveri*, *M. capitatus*, *H. opuntiae*, *W. subpelliculosus*, and *A. pullulans*, rDNA sequencing was employed to identify their species. It seems that the exact factors contributing to the emergence of rare yeast species are still unclear. However, sociodemographic variables, the impact of drugs in fostering favorable environments for yeast growth, compromised health status, and the employed identification techniques may contribute to the detection of uncommon yeasts. Importantly, unlike the studies conducted by Maheronnaghsh et al.^35^ on oral yeasts in cancer patients and Erfaninejad et al.^16^ conducted on HIV patients in southwestern Iran, our study successfully identified uncommon yeast species. Among the rare species identified in the present study, *G. capitatum* poses a risk of systemic geotrichosis in immunocompromised patients, particularly those with severe neutropenia^36^, while *C. lusitaniae* can develop multidrug resistance, leading to systemic infections^37^. Moreover, our study reported the first case of an *M. capitatus* infection in the oral cavity of an individual with a history of heroin and amphetamine abuse. In essence, there is serious concern for immunocompromised patients, given that rare yeast isolates possess the potential to infiltrate deeper tissues and induce infections under specific conditions.

The present study also revealed significant difference in the occurrence of OYC between females (80.6%) and males (62.8%). This finding diverges from the results of Suryana et al., who reported a markedly higher prevalence of oral candidiasis in males compared to females^27^. The higher occurrence of OYC in women may be attributed to various factors. Hormonal fluctuations, especially during menstruation, pregnancy, or menopause, can affect the oral environment and promote yeast growth. Additionally, the use of hormonal contraceptives may influence the oral microbiota^38–40^.

In our study, we witnessed that older age was a contributing factor to the development of OYC in individuals with SUD. All individuals aged 50 years or older exhibited colonization in contrast to 65.5% of those aged less than 50 years. Old age may be a predisposing factor for oral candidiasis, possibly due to complex systemic conditions, decreased salivary flow, increased medication intake, and denture wearing^41^.

We did not detect significant differences in the prevalence of OYC between individuals who smoked cigarettes and those who did not smoke cigarettes. However, owing to the limited number of non-cigarette users included in our study, we were unable to conclusively determine whether cigarette smoking influenced the pattern of yeast colonization in the oral cavity.

In the present study, we performed a logistic regression analysis to identify the factors associated with oral yeast candidiasis in individuals with substance abuse. Initially, a univariate logistic regression model was fitted, and variables with P-values less than 0.25 were included in the MLR model. Our analyses revealed that opioid use, oral drug use, rate of drug consumption per day, duration of drug use, and DSM-5 score were significantly associated with the occurrence of OYC. It is worth noting that drugs can be broadly classified into four main groups: opioids (tramadol, crack, heroin, opium, methadone, norjizak, and sorche), hallucinogens (hashish, grass, marijuana, and mushrooms), stimulants (methamphetamine, crystal, and cocaine), and other substances (nasvay and alcohol). To our surprise, after controlling for the effect of other variables in the model, the odds of having OYC were 3.27 times higher in patients using opioids than in those who did not use opioids. Several studies have demonstrated that opioids have suppressive effects on the immune system, which can impact cytokine production, antibody production, and immune cell migration and function^9,42,43^. In addition, chronic drug use could potentially cause structural changes to the oral mucosa and tissues, rendering them more susceptible to fungal overgrowth and colonization^44,45^.

As our results indicated, drug use was observed via various routes, including oral ingestion, injection, and smoking. The results demonstrated that individuals who reported oral drug use had 6.96 times higher odds of experiencing OYC compared to those who did not report oral drug use. One possible explanation for this association is that oral drugs may cause dry mouth as a side effect, which can disrupt the saliva’s natural defenses against OYC. Saliva contains antibodies and enzymes that help control yeasts levels^46,47^.

Furthermore, the results of our study revealed a significant association between longer duration of drug use and higher daily consumption rates with increased odds of OYC. There are various factors that may contribute to this association. Firstly, chronic drug exposure may induce immunosuppression, which can increase the susceptibility to OYC. Secondly, long-term drug use may increase the likelihood of oral side effects, such as xerostomia, which can disrupt the natural defenses of the oral cavity against OYC. Finally, higher cumulative drug intake may lead to a greater disturbance of the oral microbiome, which can promote the growth of *Candida* spp. and other microorganisms^10,48^.

The Diagnostic and Statistical Manual of Mental Disorders, Fifth Edition (DSM-5) is a standard classification system for mental disorders developed by the American Psychiatric Association^49,50^. In our study, we found that the DSM-5 score was negatively associated with OYC, with an odds ratio of 0.91. One possible explanation is that patients with severe SUD may be less compliant with oral hygiene routines due to their addiction, which could increase their risk of developing OYC. Additionally, individuals with more entrenched addictions may spend less time focusing on oral health symptoms, leading to underreporting of OYC in the study. These factors may contribute to the lower odds of OYC among individuals with higher DSM-5 scores. Nevertheless, further research is imperative to validate these explanations and unveil the underlying mechanisms of this association.

## Conclusion

The present study revealed a heightened prevalence of oral yeasts, notably uncommon yeast species, in individuals with substance use disorders. Additionally, factors such as the duration of drug use, daily drug consumption, DSM-5 score, opioid use, and oral drug administration were significantly associated with an elevated likelihood of oral yeast colonization. These findings highlight the multifaceted etiology, primarily influenced by drug-related factors that compromise immunity and impact saliva flow and composition. For a more comprehensive understanding of the oral yeast diversity in this high-risk population, it is recommended to carry out further prospective multicenter studies in different geographical regions through employing reliable diagnostic methods.

### Supplementary Information

Supplementary Figures.

## Data Availability

The data that supports the findings of this study are available in the supplementary material of this article.
