# Supplementary material for: Exploring the diversity of uncommon oral yeast species and associated risk factors among substance abusers in southwestern Iran
Source: Sci Rep. 2024 Jan 22;14:1906. doi: 10.1038/s41598-024-52105-4 (PMC10803760; doi:10.1038/s41598-024-52105-4)
Supplement: Supplementary file 1 — Supplementary Figures. [file 41598_2024_52105_MOESM1_ESM.doc]

**Supplementary figures legends**

**
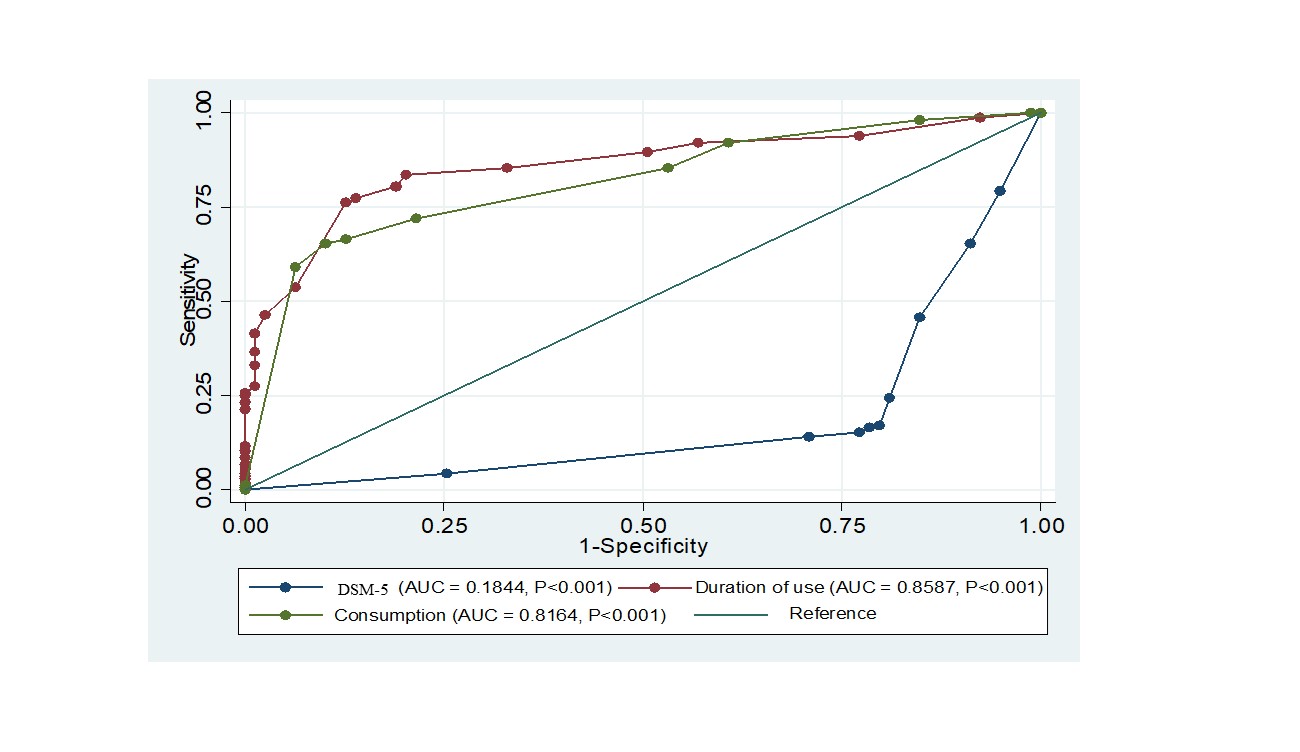
**

**Figure S1.** Comparison of the predicting power of having OYC by the variables of duration of use, consumption, and DMSIV using the ROC curve obtained by fitting the univariate LR model.


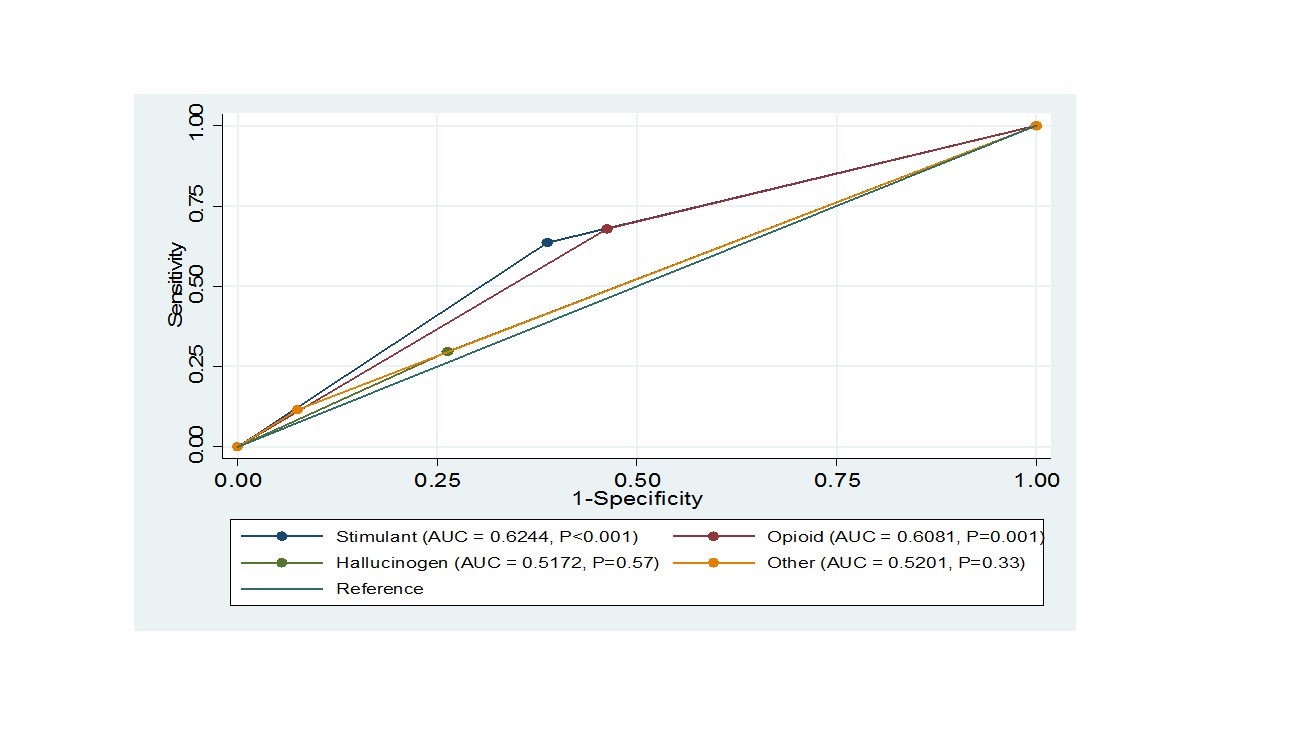


**Figure S2.** Comparison of the predicting power of having OYC by the variables of consumption of stimulant, hallucinogen, opioid and other substances using the ROC curve resulting from the fitting of the univariate LR model.

**
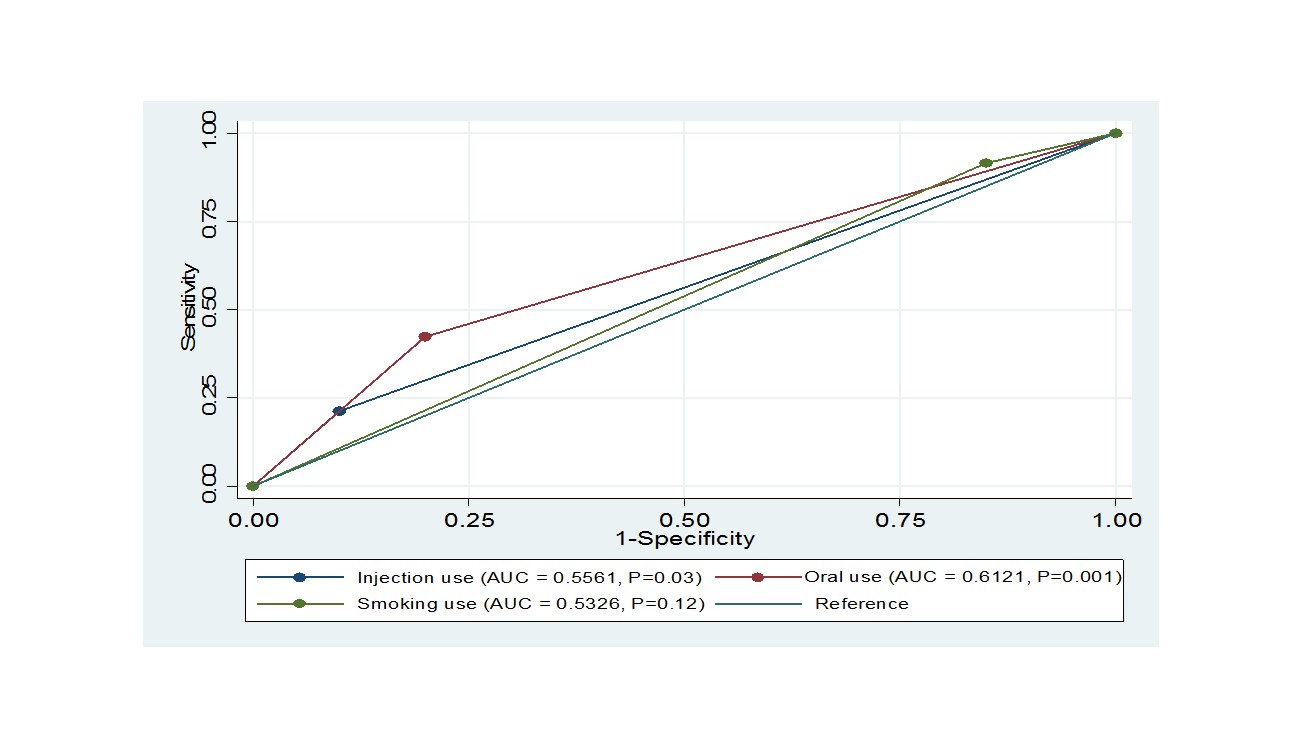
**

**Figure S3.** Comparison of the predicting power of having OYC by variables of substance use consumption using the ROC curve resulting from the fitting of the univariate LR model.
